# Acute Kidney Injury Following Whole Cherry Consumption as a Sleep Aid in a Geriatric Patient With Chronic Kidney Disease

**DOI:** 10.7759/cureus.117831

**Published:** 2026-10-12

**Authors:** Kourosh A Moshiri, Luke Okamuro, Jay Lee

**Affiliations:** 1 Geriatrics/Internal Medicine, Eisenhower Medical Center, Rancho Mirage, USA; 2 Geriatrics/Family Medicine, Eisenhower Medical Center, Rancho Mirage, USA; 3 Geriatrics, Eisenhower Medical Center, Rancho Mirage, USA

**Keywords:** acute kidney injury, cherry, chronic kidney disease (ckd), nutraceuticals, sleep aids

## Abstract

Cherries are consumed for their anti-inflammatory and sleep-promoting effects, but their potential adverse effects remain poorly characterized. A 95-year-old woman with stage 3A chronic kidney disease (CKD) presented with an increase in serum creatinine from a baseline of 0.8 to 1.3 mg/dL and a decrease in estimated glomerular filtration rate from 55.3 to 35.2 mL/min/1.73 m² following two weeks of consuming 15-20 fresh or frozen whole cherries nightly. Cherry consumption was discontinued, and her kidney function returned to baseline one month later. Cherries contain high amounts of anthocyanins, which may contribute to acute kidney injury (AKI) in CKD patients through cyclooxygenase (COX)-inhibitory mechanisms, similar to nonsteroidal anti-inflammatory drugs (NSAIDs). Application of the Naranjo Adverse Drug Reaction Probability Scale yielded a score of 7, indicating a "probable" association between cherry consumption and AKI.

## Introduction

Cherries are sometimes consumed as a nutraceutical for their established anti-inflammatory and sleep-promoting benefits. Both sweet cherries, such as Bing, and tart cherries, such as Montmorency, contain compounds including anthocyanins, other polyphenols, and melatonin, although the concentrations of these compounds vary depending on cultivar and preparation [1]. Their melatonin and polyphenol content has led to growing interest in their use as a natural sleep aid and for their potential anti-inflammatory effects. Tart cherry products have been studied for possible sleep-promoting effects, with some studies reporting improvements in sleep duration, sleep efficiency, or sleep onset. However, the available clinical evidence remains limited, with considerable variation in study populations, cherry preparations, doses, and measured sleep outcomes [2]. Despite their reputation as natural and generally healthful, cherry-derived products may also pose risks to susceptible individuals. Previous case reports have described acute kidney injury (AKI) following daily consumption of cherry juice concentrate in older adults with pre-existing chronic kidney disease (CKD), with kidney function improving after discontinuation [3,4]. This report describes reversible AKI associated with whole cherry consumption as a sleep aid, extending previous reports involving concentrated cherry products.

## Case presentation

A 95-year-old woman with a history of hypertension, hyperlipidemia, stage 3A CKD, osteoarthritis, and lumbar spondylosis with radiculopathy presented for routine follow-up without complaints. Her medications included atorvastatin 10 mg, calcium citrate with vitamin D3, cyanocobalamin 250 mcg, diltiazem CD 300 mg, gabapentin 100 mg, and lisinopril 20 mg. Her medication regimen was reviewed and had remained unchanged. She denied any recent illness, fever, lower urinary tract symptoms (LUTS), gastrointestinal symptoms, changes in fluid intake, or use of over-the-counter analgesics, including NSAIDs (nonsteroidal anti-inflammatory drugs). Physical examination was unremarkable, with no signs of volume depletion.

Laboratory studies revealed AKI superimposed on CKD, with an increase in serum creatinine from a baseline of 0.8 mg/dL to 1.3 mg/dL and a decrease in estimated glomerular filtration rate (eGFR) from 55.3 to 35.2 mL/min/1.73 m². Serum potassium was within normal limits. Urinalysis was bland, without proteinuria, hematuria, or cellular casts. The remainder of her laboratory studies were unremarkable. Further history revealed that the only recent change was the nightly consumption of 15-20 fresh or frozen whole cherries for two weeks, taken as a sleep aid after reading online that cherries could promote sleep. Cherry consumption was discontinued. Repeat laboratory testing one month later showed that serum creatinine had returned to baseline at 0.9 mg/dL and eGFR had improved to 52.31 mL/min/1.73 m² within her baseline range.

To formally assess the likelihood that cherry consumption contributed to the observed AKI, the Naranjo Adverse Drug Reaction Probability Scale [5] was applied (Table 1). The total score in this case was 7 out of a possible 13, corresponding to a "probable" adverse drug reaction (score 5-8) as described in Table 1.

**Table 1:** Naranjo Adverse Drug Reaction Probability Scale Applied to Cherry-Associated AKI Score interpretation: ≥9 = Definite; 5–8 = Probable; 1–4 = Possible; ≤0 = Unlikely

Question	Answer	Score
1. Are there previous conclusive reports of this reaction?	Yes	+1
2. Did the adverse event appear after the suspected agent was administered?	Yes	+2
3. Did the adverse reaction improve when the agent was discontinued?	Yes	+1
4. Did the adverse reaction reappear when the agent was re-administered?	Not done	0
5. Are there alternative causes that could have caused the reaction?	No	+2
6. Did the reaction appear when a placebo was given?	Not applicable	0
7. Was the drug detected in the blood in toxic concentrations?	Not done	0
8. Was the reaction more severe with increased dose or less severe with decreased dose?	Not done	0
9. Did the patient have a similar reaction to the same or similar agent previously?	No	0
10. Was the adverse event confirmed by objective evidence?	Yes	+1
Total Score	-	7 (Probable)

## Discussion

To our knowledge, this is the first reported case of a reversible decline in kidney function observed after consuming whole cherries.

Key contributors to the Naranjo score included the temporal relationship between cherry consumption and AKI onset (+2), prior published reports of the same reaction with cherry products (+1) [3,4], improvement in AKI upon discontinuing the therapy (+1), objective laboratory confirmation (+1), and no alternative causes identifiable (+2). Several alternative causes of AKI were considered. The patient's medication regimen, including lisinopril 20 mg, had been stable for an extended period without prior changes in kidney function, making a new drug-related effect unlikely. There were no signs or symptoms of volume depletion, LUTS, current illness, or urinary obstruction. She denied use of NSAIDs or other over-the-counter analgesics. Urinalysis was unremarkable, without evidence of urinary tract infection (UTI), glomerulonephritis, or acute tubular necrosis.

Key limitations to the score include the absence of anthocyanin serum levels and dose-response data due to the unavailability of biomarker assays, which is a recognized limitation when applying the Naranjo scale to dietary supplements [6]. The rechallenge test was also not performed, as it would not have been clinically appropriate.

A possible mechanism to explain the adverse renal effects of cherries involves the anthocyanins and other phenolic compounds found in cherries, which have demonstrated cyclooxygenase (COX)-inhibitory activity in laboratory studies [7,8]. COX enzymes contribute to the production of vasodilatory renal prostaglandins, including prostaglandin E2 and prostacyclin, which help preserve afferent arteriolar blood flow when renal perfusion is compromised [9,10]. Reduced prostaglandin production may limit this protective vasodilation, decreasing blood flow to the glomerulus, intraglomerular pressure, and glomerular filtration. This proposed mechanism resembles the hemodynamic kidney injury associated with NSAIDs, in which COX inhibition has minimal impact on individuals with adequate renal reserve but may become clinically significant when renal perfusion is already compromised [10]. The patient's advanced age and underlying CKD may have increased her susceptibility, as aging is associated with frailty, nephron loss, and reduced renal reserve, limiting the kidney's ability to compensate for additional hemodynamic stress [11,12].

The two previously published cases involved AKI associated with concentrated cherry juice. Luciano [3] described AKI and hyperkalemia in an older patient with stage 3 CKD following daily cherry concentrate consumption. Kidney function improved after the concentrate was discontinued, although lisinopril and furosemide were also temporarily withheld, introducing potential confounders. Matout et al. [4] reported AKI following daily black cherry concentrate consumption in an older patient with advanced CKD who was receiving several medications that could affect renal hemodynamics. In contrast, our patient consumed whole cherries, and, other than discontinuing her cherry consumption, her medication regimen remained unchanged. The absence of confounding medication changes in the present case strengthens the association and is reflected in the Naranjo score. The recovery of kidney function following discontinuation in all three cases supports consideration of cherry exposure as a possible contributor. However, differences in the form, quantity, and duration of cherry intake make it difficult to determine which exposures may present clinically meaningful risk.

Several limitations should be acknowledged. The exact cherry variety was not documented, and intake was reported by number rather than weight. COX activity, prostaglandin levels, and renal blood flow were not measured; therefore, the proposed mechanism could not be directly evaluated. The Naranjo scale, while widely used, was originally designed for pharmaceutical agents and has inherent limitations when applied to dietary products, including the inability to measure serum drug levels or assess dose-response relationships for food-derived compounds. Other causes of the change in kidney function cannot be entirely excluded, although a thorough evaluation did not identify an alternative etiology.

## Conclusions

This case of a 95-year-old woman with CKD developing reversible AKI after consuming whole cherries for two weeks as a sleep aid, with a Naranjo score of 7 ("probable"), emphasizes that natural foods used for therapeutic purposes may carry unappreciated risks. This single case does not establish causality, and the proposed mechanism remains unconfirmed.

Nutraceuticals warrant the same benefit-risk assessment as conventional pharmacotherapy. Clinicians evaluating unexplained kidney dysfunction should inquire about changes in medications, supplements, and diet as part of history gathering. Frequent use of cherries as a sleep aid may warrant caution in patients with underlying CKD.
